# Imaging Liver Development/Remodeling in the See-Through Medaka Fish

**DOI:** 10.1186/1476-5926-2-S1-S30

**Published:** 2004-01-14

**Authors:** David E Hinton, Yuko Wakamatsu, Kenjiro Ozato, Shosaku Kashiwada

**Affiliations:** 1 Division of Environmental Sciences and Policy, Nicholas School of the Environment and Earth Sciences, Duke University, Durham, North Carolina 27708-0328, USA; 2 Laboratory of Freshwater Fish Stocks, Bioscience Center, Nagoya University, Nagoya, Japan

## Introduction

One major function of the livers of fishes, essential for life, is the metabolism of xenobiotics, rendering lipophilic compounds water soluble and more easily excreted. This function is in turn linked to the formation and excretion of bile. Linked hepatocytic and biliary epithelial function in fishes involves hepatic tubules [1-3]. In these, hepatocytes are clustered about an axis of the biliary system. This pattern is analogous to that of an exocrine gland and is the adult phenotype for amphibians, birds, reptiles, and fishes [3].

Most fish species lack resident macrophages of the hepatic sinusoids (Kupffer cells) [3]; and, while mammalian studies – including recent findings reported at this meeting – clearly show important roles of these and other cells of the hepatic sinusoids in initiation and modulation of hepatoxic responses, studies in fishes lag far behind those of their mammalian counterparts. Our overall objective is to improve understanding of the biology of laboratory model fish so that future workers may have better tools with which to approach the complexity of environmental science including toxicology. Herein, we present, for the first time, results of *in situ *and vital studies on liver development, metamorphosis and formation of adult vascular elements in medaka. These studies were greatly facilitated by use of the see-through medaka [4], a vertebrate model with a transparent body throughout life.

## Methods

Individual ST II (see-through) medaka (*Oryzias latipes*) were used. Production of these mutants is described by Wakamatsu et al. [4]. From about 50 natural color mutants of medaka in the Laboratory of Freshwater Fish Stocks, Bioscience Center, Nagoya University (Nagoya, Japan) [5,6], some were selected that showed deficiency in pigmentation. By crossing selected mutants, Wakamatsu, et al. [4] genetically removed pigments from the entire body, thereby generating a transparent fish. Breeding groups of ST II medaka fed a commercial ration (Otohime Beta, Nisshin Feed Co. Ltd., Tokyo) twice daily and supplemented with brine shrimp *nauplii *for four days each week were maintained under a 16:8 hr light/dark cycle at 26–C (spawning conditions). Fertilized eggs were collected daily and development recorded through the transparent chorion using a dissection microscope (Leica Wild M420; equipped with a Nikon 990 cool pix camera). To facilitate orientation and to improve imaging of certain embryonic stages, dechorionation was employed using medaka hatching gland enzyme as described [7,8]. Embryonated eggs of other medaka were collected weekly for 12 wks. Iwamatsu [9] described and figured developmental stages of medaka and this was followed to select liver development from early organogenesis, through larval and juvenile stages into spawning females. Specimens for high-resolution light microscopy (HRLM) were directly placed in fixative (embryos) or were first killed by overdose in anesthetic (tricaine methane sulfonate; larvae and older) then immediately placed in fixative (10% neutral buffered formalin or 4% paraformaldehyde in phosphate buffer). After 72 hrs, fixed specimens were transferred to phosphate buffered saline containing 6% sucrose, stored in the cold until time of processing, embedded in glycol methacrylate, sectioned at 2–4 microns thickness, stained by Gill's hematoxylin and eosin and viewed using a Nikon Eclipse E600 binocular microscope with digital still camera system (DXM 1200).

## Results

**Figure 1:**
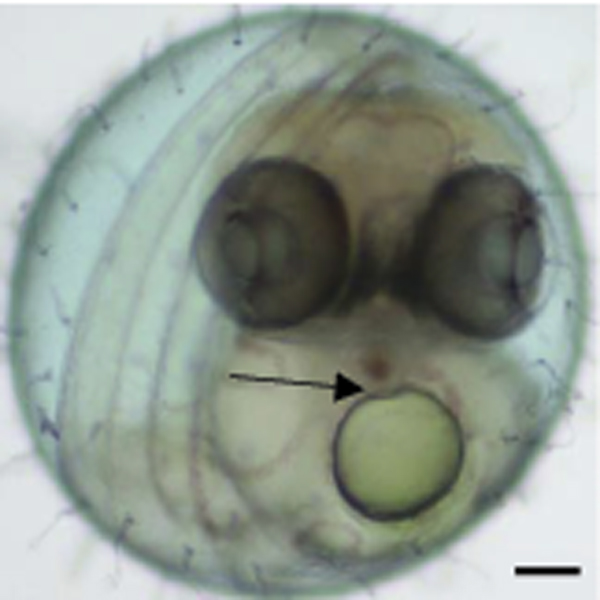
Stage 37 living medaka embryo showing mandible between eyes, forebrain, heart under mouth and lft. & rt. ducts of Cuvier. Contraction of ventricle deforms oil droplet of yolk sac (arrow). Bar = 0.1 mm.

**Figure 2:**
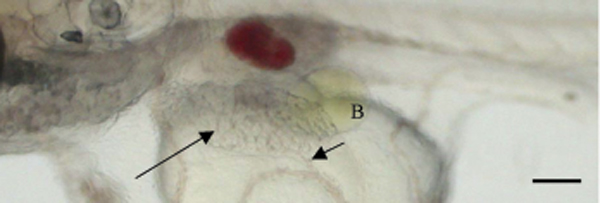
Long arrow points to liver. Short arrow indicates left hepatic vein conducting blood from liver to left duct of Cuvier. Note pigment in gall bladder (B). Head is positioned to the left and dorsal surface is at top of figure. In left upper corner note otolith in inner ear. Bar = 0.1 mm.

Imaging of the living ST II medaka at stage 37 [9] is shown in Figure (1). The chorion (eggshell) was intact and had numerous small filaments on outer surface. Examination of the chorionated embryos was made complex due to the rounded confines of the embryonic space. When head was viewed from rostral end with embryo in anatomic position, the caudal peduncle curved and the more distal portions of the tail extended alongside the embryo and over the head with the tip of the tail reaching a point caudal to the otic vesicle (not shown in figure). At this time, the yolk sac is large and major veins pass in circuitous routes over the yolk sac and eventually converge at the sinus venosus. After dechorionation, intra-vital analysis of embryos near normal time of hatching (Stage 39), revealed topography and anatomical features of key abdominal organs (Fig. 2) as well as inner ear. Spleen was visible as a red disc lateral to the swim bladder (adjacent brown disc). The elongated liver was located within the left lateral portion of the abdominal cavity (Fig. 2). The intrahepatic circulation in stage 39 was visible and a single left hepatic vein conducted blood from the liver to the left duct of Cuvier that was visible as a wavy line on the yolk sac.

**Figure 3:**
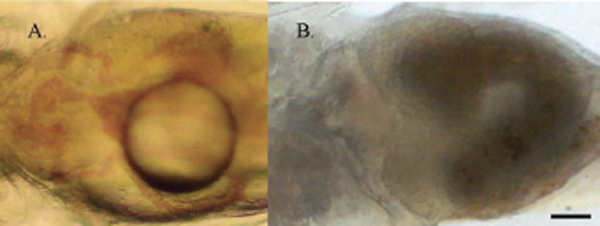
**A and B. **In 3A, from top to bottom in this ventral view of larval medaka during first wk after hatching, are the 3 major veins associated with yolk sac and abdominal viscera: left duct of Cuvier, median yolk vein (deep to oil droplet) and right duct of Cuvier. Note that the liver (asterisk) remains in the left lateral portion of the abdominal cavity. In 3B, a ventral view of 2 wk old hatchling, note absence of major veins at the abdominal surface. Liver has now assumed a transverse position extending across the full extent of the rostral portion of abdomen. Heart now occupies concavity associated with rostral liver surface. Bar = 0.1 mm.

**Figure 4:**
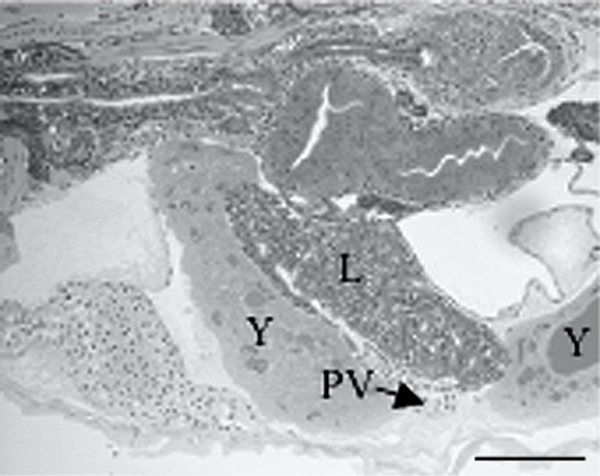
GMA sagittal section of larval medaka at wk 2 after hatch. Liver (L) has incorporated median yolk vein as hepatic portal vein (PV). Only remnants of yolk sac persist (Y). Pneumatic duct attaches swim bladder to esophagus. Bar = 0.1 mm.

Remodeling of liver, major veins of yolk sac and abdomen was apparent when larvae of wk one after hatching were compared to those one week older (wk 2). When the liver was in the left lateral portion of the abdominal cavity, ducts of Cuvier and median yolk vein were apparent in ventral view of abdomen (Fig. 3A). One week later, a similar view revealed metamorphosis of liver. Position of liver was now transverse and location was in the rostral most portion of the abdominal cavity (Fig. 3B). Also, left and right ducts of Cuvier were not imaged and there was no indication of the median yolk vein. When medaka at this time of development were processed for HRLM, sagittal sections revealed incorporation of median yolk vein into liver (Fig. 4).

**Figure 5:**
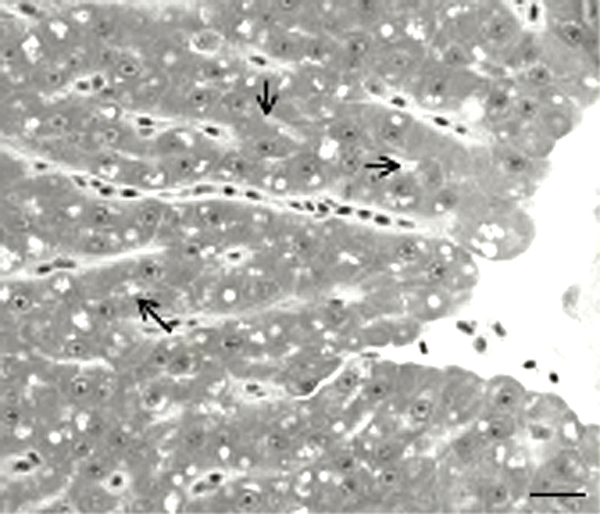
GMA section of liver from actively spawning female medaka. Hilus of liver is at right of field. Hepatic tubules are in near linear array and biliary space is in midtubular location. (Arrows). Bar = 10 –m.

The adult pattern of hepatic tubular architecture was most apparent in actively spawning female medaka at 8 wks of age (Fig. 5). Hepatocytes were arranged as tubules with biliary lumens in center.

## Discussion

Use of see-through medaka greatly facilitates correlation of liver development, metamorphosis and maturation. Pigment interference is not a problem in this model. Medaka are being used to investigate endocrine disruptors [10,11], liver carcinogenesis [12,13] and the efficiency of new waste water treatment and reuse systems (unpublished studies this laboratory). Their small size has led to their incorporation in studies in outer space as well. Their usefulness will be greatly enhanced following improved understanding of the roles of various liver cell types in health and disease.
